# TRPC6-dependent Ca^2+^ signaling mediates airway inflammation in response to oxidative stress via ERK pathway

**DOI:** 10.1038/s41419-020-2360-0

**Published:** 2020-03-05

**Authors:** Qingzi Chen, Yubo Zhou, Lifen Zhou, Zhaodi Fu, Chuntao Yang, Lei Zhao, Shuni Li, Yan Chen, Yousen Wu, Zhenwei Ling, Yufeng Wang, Jianrong Huang, Jianhua Li

**Affiliations:** 1 Affiliated Cancer Hospital & Institute; Key Laboratory of Protein Modification and Degradation, School of Basic Medical Sciences, Guangzhou Medical University, Guangzhou, China; 2 Institute of Pediatrics, Guangzhou Women and Children’s Medical Center of Guangzhou Medical University, Guangzhou, China; 3 The Fifth Affiliated Hospital of Guangzhou Medical University, Guangzhou, China

**Keywords:** Ion channels, Molecular biology

## Abstract

Ozone (O_3_) plays an extremely important role in airway inflammation by generating reactive oxygen species (ROS) including hydrogen peroxide, then promoting redox actions and causing oxidative stress. Evidences indicate that TRPC6 (canonical transient receptor potential channel 6) is a redox-regulated Ca^2+^ permeable nonselective cation channel, but its role in the setting of oxidative stress-related airway inflammation remains unknown. Here, we found that both TRPC6^−/−^ mice and mice pretreated with SAR7334, a potent TRPC6 inhibitor, were protected from O_3_-induced airway inflammatory responses. In vitro, both knockdown of TRPC6 expression with shRNA and TRPC6 blockage markedly attenuated the release of cytokines IL-6 and IL-8 induced by O_3_ or H_2_O_2_ in 16HBE cells (human bronchial epithelial cell line). Treatment with O_3_ or H_2_O_2_ enhanced TRPC6 protein expression in vivo and vitro. We also observed that TRPC6-dependent increase of intracellular Ca^2+^ concentration ([Ca^2+^]_i_) was triggered by H_2_O_2_, which consisted of the release from intracellular calcium store and the influx of extracellular Ca^2+^ and could be further strengthened by 6-h O_3_ exposure in both 16HBE cells and HBEpiCs (primary human bronchial epithelial cells). Moreover, we confirmed that the activation of MAPK signals (ERK1/2, p38, JNK) was required for the inflammatory response induced by O_3_ or H_2_O_2_ while only the phosphorylation of ERK pathway was diminished in the TRPC6-knockdown situation. These results demonstrate that oxidative stress regulates TRPC6-mediated Ca^2+^ cascade, which leads to the activation of ERK pathway and inflammation and could become a potential target to treat oxidative stress-associated airway inflammatory diseases.

## Introduction

Abnormal airway inflammation resulting from exposure to various oxidizing ambient pollutants is one of the most common and significant pathogenesis for numerous respiratory diseases, including asthma, chronic obstructive pulmonary disease (COPD), and lung cancer. Ambient pollutants, such as inhalable dusts, particulate matter (PM), tobacco smoke and ozone (O_3_), have strong ability to generate reactive oxygen species (ROS), accelerate redox actions and trigger oxidative stress. As the first line of defense and major target of inhaled harmful environmental pollutants, bronchial epithelium produces a series of pro-inflammatory molecules and recruits inflammatory cells into interstitium and airways after suffering oxidative stress, which could further aggravate airway inflammation. However, the molecular mechanisms by which oxidative air pollutants trigger pulmonary inflammation are still elusive.

Ca^2+^, an essential secondary messenger relevant to a variety of cellular processes, plays a key role in mediating airway inflammatory responses. Rises in [Ca^2+^]_i_ in pulmonary cells are essential for the activation of inflammatory signal transduction proteins and transcriptions factors^1–3^. Dysregulation of [Ca^2+^]_i_ homeostasis in bronchial epithelia contributes to pulmonary disease^4–6^. In addition, ROS has been found to be responsible for the activity of various calcium channels^7^. TRPM2, a plasma membrane Ca^2+^-permeable channel, mediates ROS-induced chemokine production in monocytes^8^. Compared with WT mice, TRPM2^−/−^ mice exhibits enhanced gastric inflammation after infecting with Helicobacter pylori, which is owing to intracellular calcium overloading and augmented oxidative stress^9^. These findings suggest that the abnormality of [Ca^2+^]_i_ suffered from ROS in pulmonary cells may be involved in airway inflammation.

TRPC6, a Ca^2+^-permeable non-selective cation channel of the canonical transient receptor potential (TRPC) family, is widely expressed in a number of tissues including brain, heart, lung, ovary, kidney, and vascular tissues^10^. Consistent with its broad expression in lungs, including bronchial epithelial cells, alveolar macrophages and pulmonary vasculature^11–13^, TRPC6 contributes to pulmonary disorders, such as cystic fibrosis, asthma, pulmonary hypertension, COPD, lung edema, and lung fibrosis^11,14,15^. Via analyzing the TRPC6 gene promoter of pulmonary artery smooth muscle cells from patients with idiopathic pulmonary arterial hypertension (IPAH), three single-nucleotide polymorphisms are identified and one of them are found to increase basal gene promoter activity, which may link abnormal transcription of TRPC6 to the activation of NF-κB and lead to upregulated risk of IPAH^16^. The expression of TRPC6 mRNA in alveolar macrophages isolated from COPD patients is significantly more than healthy controls^12^. Particularly, as a modulator of membrane calcium currents, TRPC6 is newly considered as an essential element in the regulation of inflammatory response^17^. TRPC6 channels have been reported to regulate CXCR2-related chemotaxis via mediating calcium supply^18^. After the activation of TLR4 and generation of DAG, TRPC6-dependent Ca^2+^ influx into endothelial cells is triggered and cooperated in endotoxin-induced lung inflammation^19^. Moreover, growing evidence points out that TRPC6 acts as a redox-related channel, while the definite relation between TRPC6 and ROS seems to be affected by cell specific difference^20–23^. Recently, we reported that TRPC6 is a key element in the regulation of adhesion of neutrophils to bronchial epithelial cell with O_3_ exposure^24^, while the role and regulatory mechanisms of TRPC6 channel in oxidative stress-induced airway inflammation are still unclear.

Here, we investigated the relevance of TRPC6 in O_3_-induced airway inflammation in mice and inflammatory response in bronchial epithelial cells. We further explored the involved underlying mechanisms to extrapolate the potential of TRPC6 as target to treat oxidative stress-associated airway inflammation.

## Results

### TRPC6 is required for O_3_-induced airway inflammatory responses in mice

**Fig. 1:**
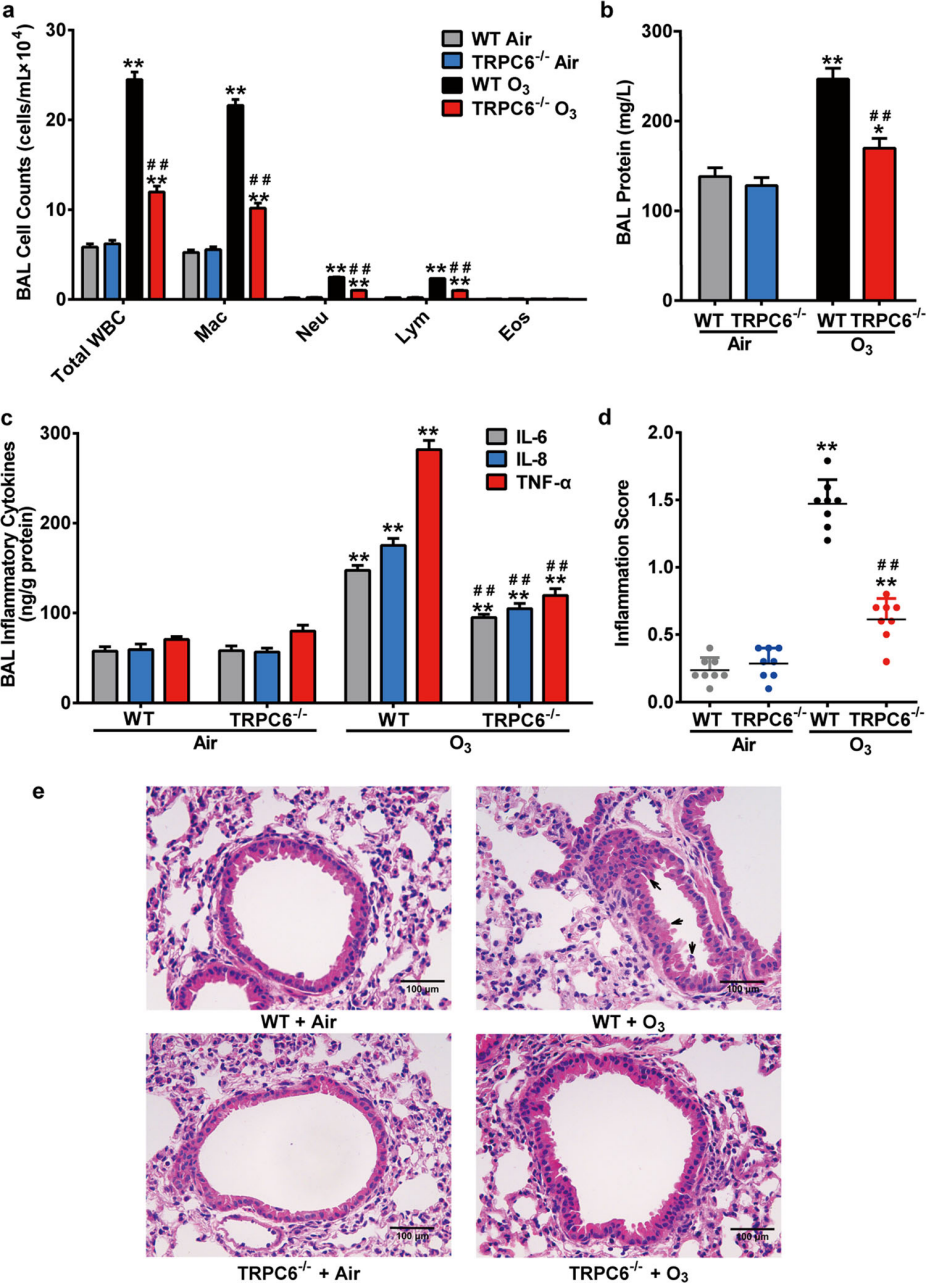
Effect of TRPC6-deficiency on O_3_-induced airway inflammation. WT and TRPC6^−/−^ mice were exposed to O_3_ (1 ppm) for 3 h every other day (day 1, 3, 5).The mice were anesthetized 24 h after the last exposure. **a**–**c** Total white blood cell counts (Total WBC), macrophage counts (Mac), neutrophil counts (Neu), lymphocyte counts (Lym), eosinophil counts (Eos) (**a**), total protein content (**b**) and the release of inflammatory mediators IL-6, IL-8, TNF-α (**c**) in BAL fluid of different groups were compared. **d** Inflammation scores in air control and O_3_-exposed mice. **e** Representative histological sections of mouse lungs (H&E staining) after exposure to O_3_ or air. Black arrows: inflammatory changes. Scale bar: 100 μm. Results are presented as mean ± SEM, *n* = 8. *P < 0.05 or **P < 0.01 compared with WT + Air group, ^##^*P* < 0.01 compared with WT + O_3_ group.

**Fig. 2:**
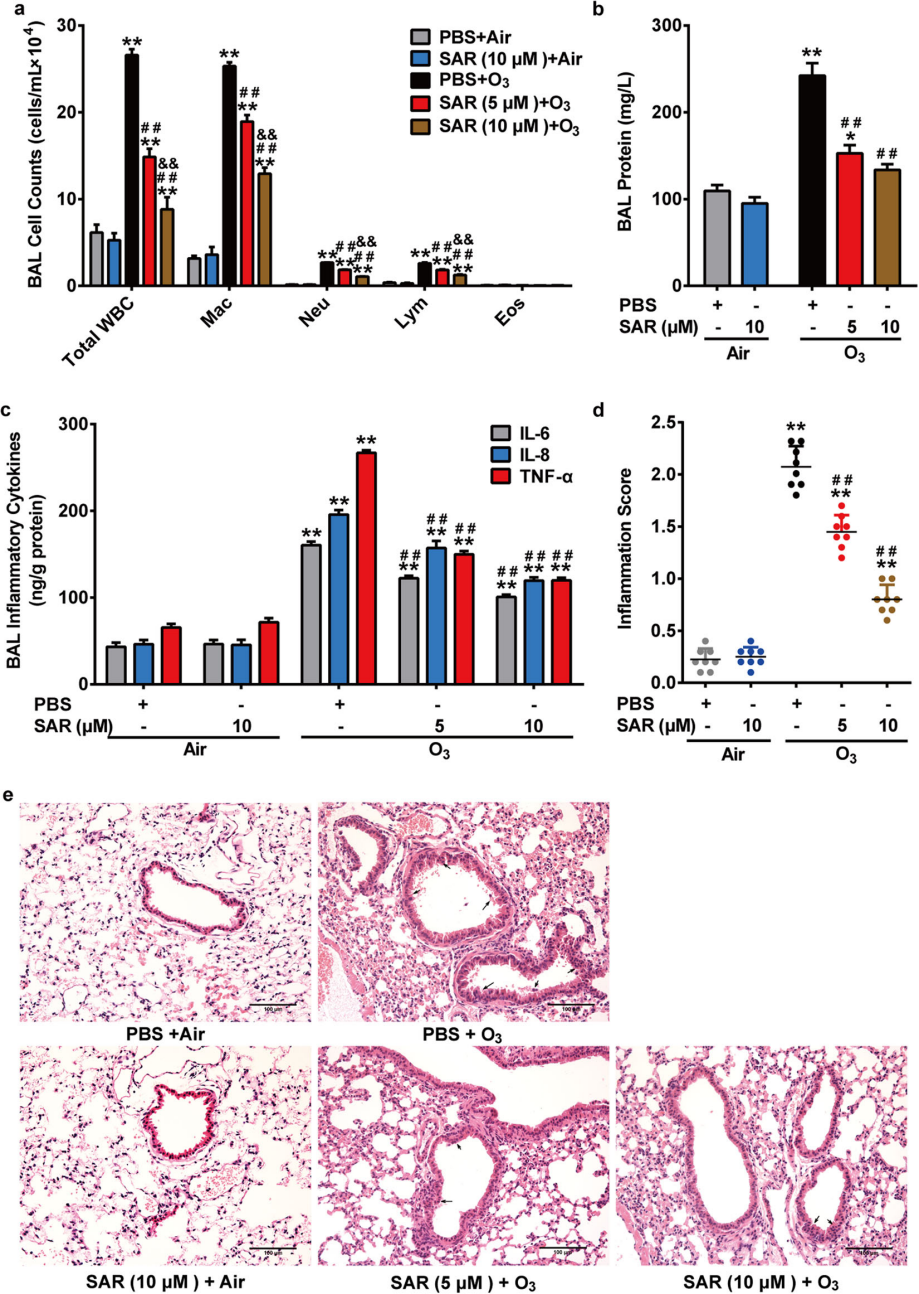
Effect of TRPC6-blockage on O_3_-induced airway inflammation in mice. WT mice were exposed to O_3_ (1 ppm) for 3 h every other day (day 1, 3, 5). Mice received PBS or SAR7334 by oral gavage 4 h before exposure. The mice were anesthetized 24 h after the last exposure. **a**–**c** Total white blood cell counts (Total WBC), macrophage counts (Mac), neutrophil counts (Neu), lymphocyte counts (Lym), eosinophil counts (Eos) (**a**), total protein content (**b**) and the release of inflammatory mediators IL-6, IL-8, TNF-α (**c**) in BAL fluid of different groups were compared. **d** Inflammation scores in air control and O_3_-exposed mice. **e** Representative histological sections of mouse lungs (H&E staining) after exposure to O_3_ or air. Black arrows: inflammatory changes. Scale bar: 100 μm. Results are presented as mean ± SEM, *n* = 8. *P < 0.05 or **P < 0.01 compared with PBS + Air group, ^##^*P* < 0.01 compared with PBS + O_3_ group, ^&&^*P* < 0.01 compared with SAR (5 μM) + O_3_ group.

We sought to investigate the function of TRPC6 in O_3_-induced airway inflammatory response by employing TRPC6^−/−^ mice and SAR7334, a TRPC6-selective inhibitor. As shown in Figs. 1, 2, both TRPC6^−/−^ mice and mice pretreated with SAR7334 failed to respond to O_3_ exposure entirely as exhibiting mild airway inflammatory response. TRPC6-deficiency as well as SAR7334 significantly inhibited O_3_-induced inflammatory cell recruitment in BAL fluid, as reflected by the reduced numbers of neutrophils, macrophages and lymphocytes but not eosinophils, compared with that in WT + O_3_ group or PBS + O_3_ group (Figs. 1a, 2a). Not only TRPC6^−/−^ mice but also mice pretreated with SAR7334 had lower total protein, IL-6, IL-8, and TNF-α content in BAL fluid than WT mice had after O_3_ exposure (Figs. 1b, c, 2b, c). The O_3_-induced increased lung inflammation scores and inflammatory changes of lung sections were also significantly inhibited by TRPC6-deficiency or SAR7334 (Figs. 1d, e, 2d, e). Given the above, TRPC6 contributes to the development of O_3_-induced airway inflammation in mice.

### Ca^2+^ signal and TRPC6 is required for oxidative stress-induced inflammatory responses in human bronchial epithelial cells

**Fig. 3:**
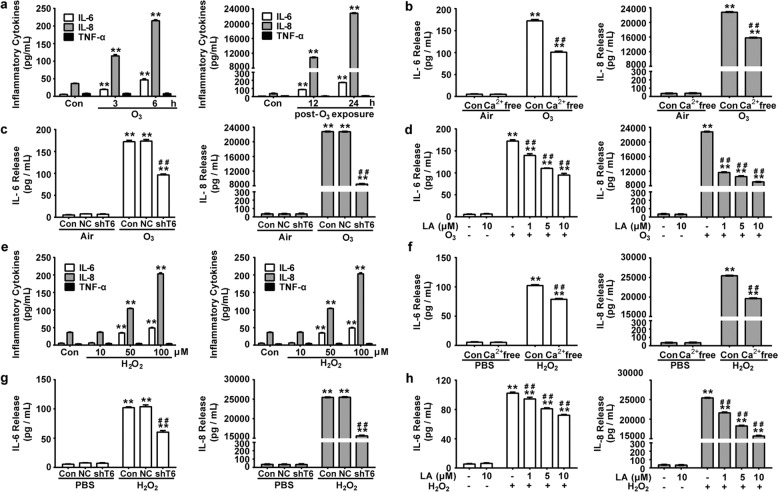
Role of Ca^2+^ and TRPC6 in oxidative stress-induced inflammatory responses in 16HBE cells. **a** Release levels of IL-6, IL-8, and TNF-α were detected after 16HBE cells were exposed to O_3_ (100 ppb) for 0, 3, 6 h (a, Left) or cultured in fresh atmosphere for another 12, 24 h following 6-hour O_3_ (100 ppb) exposure (**a**, Right). Data represent the mean ± SEM, *n* = 5. **P < 0.01 compared with the Control group. **b** 16HBE cells incubated with or without Ca^2+^-free bath solution containing 100 μM EGTA were exposed to O_3_ (100 ppb) for 6 h and then cultured in fresh atmosphere for another 24 h. Release levels of IL-6 and IL-8 were detected. Data represent the mean ± SEM, *n* = 5. **P < 0.01 compared with Control group, ^##^*P* < 0.01 compared with O_3_ group. **c** After NC or shTRPC6 infection, 16 HBE cells were exposed to O_3_ (100 ppb) for 6 h and then cultured in fresh atmosphere for another 24 h. Release levels of IL-6 and IL-8 were detected. Data represent the mean ± SEM, *n* = 5. **P < 0.01 compared with Control group, ^##^*P* < 0.01 compared with O_3_ group, ^&&^*P* < 0.01 compared with NC + O_3_ group. Con: Control, NC^:^ Negative Control, shT6: shRNA TRPC6. **d** After pretreatment with LA (0, 1, 5, 10 μM) for 1 h, 16HBE cells were exposed to O_3_ (100 ppb) for 6 h and then cultured in fresh atmosphere for another 24 h. Release levels of IL-6 and IL-8 were detected. Data represent the mean ± SEM, *n* = 5. **P < 0.01 compared with Control group, ^##^*P* < 0.01 compared with O_3_ group. **e** Release levels of IL-6, IL-8, and TNF-α were detected after 16HBE cells were stimulat**e**d by H_2_O_2_ (0, 10, 50, 100 μM) for 12 h (**a**, Left) or H_2_O_2_ (100 μM) for 0, 3, 6, 12, 24 h (**a**, Right). Data represent the mean ± SEM, *n* = 5. **P < 0.01 compared with Control group. **f** 16HBE cells incubated with or without Ca^2+^-free bath solution containing 100 μM EGTA were exposed to H_2_O_2_ (100 μM) for 24 h. Release levels of IL-6 and IL-8 were detected. Data represent the mean ± SEM, *n* = 5. **P < 0.01 compared with Control group, ^##^*P* < 0.01 compared with H_2_O_2_ group. **g** After NC or shTRPC6 infection, 16HBE cells were exposed to H_2_O_2_ (100 μM) for 24 h. Release levels of IL-6 and IL-8 were detected. Data represent the mean ± SEM, *n* = 5. **P < 0.01 compared with Control group, ^##^*P* < 0.01 compared with H_2_O_2_ group, ^&&^*P* < 0.01 compared with NC + H_2_O_2_ group. Con: Control, NC: Negative Control, shTRPC6: shRNA TRPC6. **h** After pretreatment with or without LA (1, 5, 10 μM) for 1 h, 16HBE cells were exposed to H_2_O_2_ (100 μM) for 24 h. Release levels of IL-6 and IL-8 were detected. Data represent the mean ± SEM, *n* = 5. **P < 0.01 compared with Control group, ^##^*P* < 0.01 compared with H_2_O_2_ group.

Ahead of studying the effect of Ca^2+^ and TRPC6 in oxidative stress-induced inflammatory response, we applied O_3_ (100 ppb) exposure on 16HBE cells and found that it made no difference to cell viability when it lasted for ≤12 h (Fig. S1a). The releases of IL-6 and IL-8 increased after exposure for 6 h and further augmented till 24 h post-exposure, but the release level of TNF-α remained unchanged (Fig. 3a). The levels of O_3_-induced production of IL-6 and IL-8 were significantly attenuated by removal of extracellular Ca^2+^ (Fig. 3b), which indicated that the influx of extracellular Ca^2+^ was a key step in O_3_-induced releases of inflammatory cytokines. We surmised that TRPC6-mediated O_3_-induced influx of extracellular Ca^2+^ as it is a potent monitor of membrane calcium currents. TRPC6 mRNA and protein expression in 16HBE cells was strikingly reduced after transducing with TRPC6 shRNA (shTRPC6) while that in the non-silenced negative control (NC) shRNA group had no difference with that in the control group (Fig. S2.). In parallel, Larixyl Acetate (LA), a potent and specific blocker of TRPC6 channels^25^, was administrated at 1, 5, 10 μM concentrations. Importantly, O_3_-induced releases of IL-6 and IL-8 were significantly reduced in shTRPC6-treated or LA-treated cells (Fig. 3c, d), suggesting an involvement of TRPC6 in O_3_-induced production of IL-6 and IL-8 in 16HBE cells.

Hydrogen peroxide (H_2_O_2_), an oxidant generated during exposure to ambient oxidizing pollutants^26^, is deemed as an intermediate capable of exerting some of its biological effects owing to its diffusibility over membranes and longer half-life than most other ROS^27^. Thus, H_2_O_2_ was used in current study to further explore the mechanism regarding O_3_-induced inflammatory response. The viability of 16HBE cells was not affected by H_2_O_2_ treatment with ≤100 μM of dose and ≤24 h of time (Fig. S1b, c). Similar with O_3_ treatment, H_2_O_2_ increased the releases of inflammatory mediators IL-6 and IL-8 in a concentration-dependent and time-dependent manner but not that of TNF-α (Fig. 3e). Consistently, absence of extracellular Ca^2+^, transfection with shTRPC6 or pretreatment with LA significantly reduced the release levels of IL-6 and IL-8 evoked by H_2_O_2_ (Fig. 3f–h), which indicated that Ca^2+^ signal and TRPC6 contributed to H_2_O_2_-induced production of IL-6 and IL-8 in 16HBE cells. Together, these results suggest that TRPC6 mediates oxidative stress-induced inflammatory responses in human bronchial epithelial cells.

### TRPC6 expression is increased by oxidative stress in vivo and vitro

**Fig. 4:**
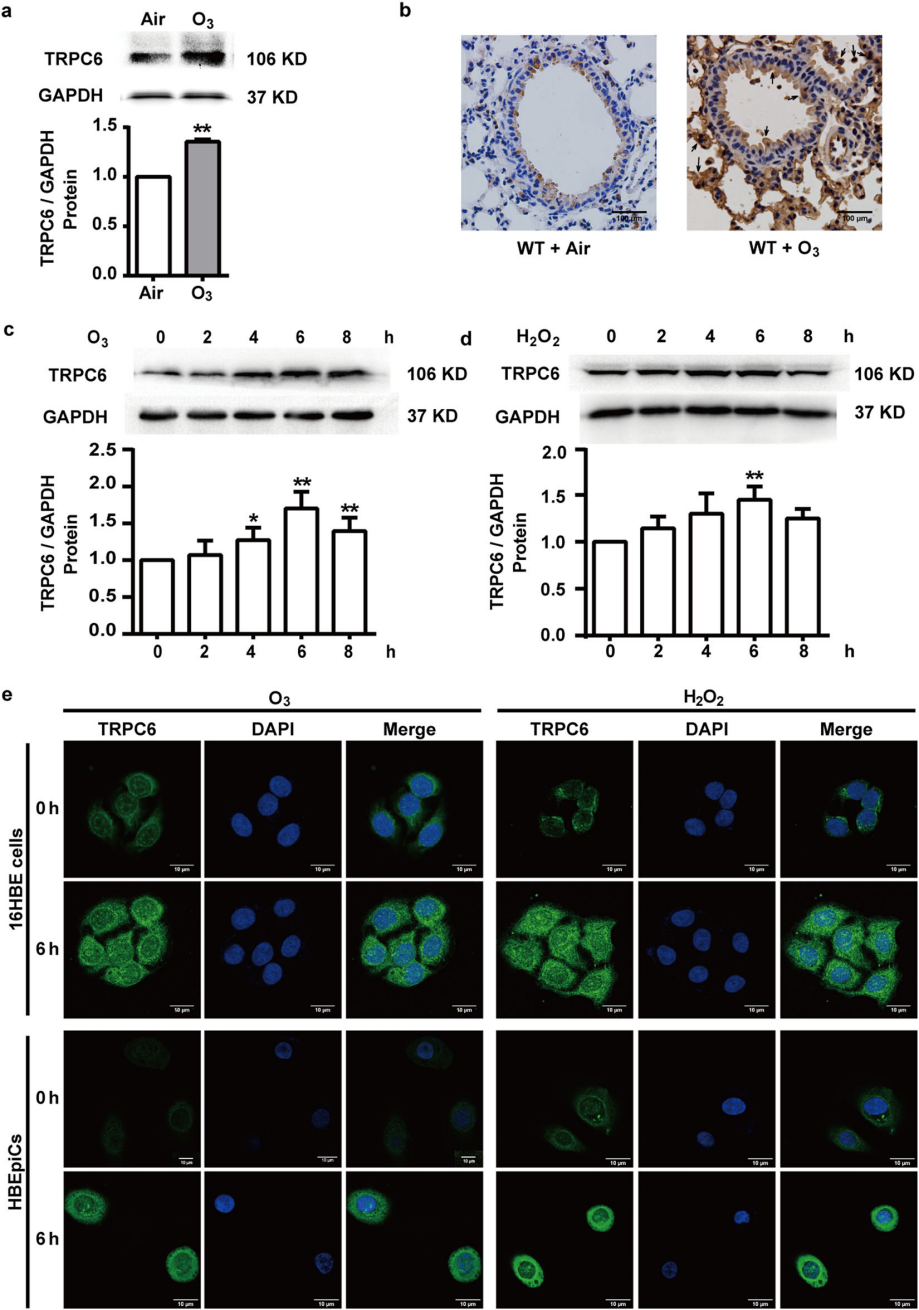
Effect of oxidative stress on TRPC6 expression in lung tissue and human bronchial epithelial cells. **a**, **b** WT mice were exposed to O_3_ (1 ppm) for 3 h every other day (day 1, 3, 5) and were anesthetized 24 h after the last exposure. TRPC6 expression in lung tissues from mice were analyzed with western blot (**a**) and immunohistochemistry-stained lung sections (**b**). Black arrows: epithelial cells and macrophages. Scale bar: 100 μm. Data represent the mean ± SEM, *n* = 8. **P < 0.01 compared with Air group. **c**, **d** TRPC6 expression in 16HBE cells was analyzed by western blot after exposure to O_3_ (100 ppb) (**c**) or H_2_O_2_ (100 μM) (**d**) for 0, 2, 4, 6, 8 h. Data represent the mean ± SEM, *n* = 5. *P < 0.05 or **P < 0.01 compared with 0 h group. **e** TRPC6 expression in 16HBE cells and primary HBEpiCs was analyzed by immunofluorescence after exposure to O_3_ (100 ppb) or H_2_O_2_ (100 μM) for 6 h. Scale bar: 10 μm. *n* = 5.

Since TRPC6-mediated oxidative stress-induced inflammatory responses, we next assessed TRPC6 protein expression in lungs of O_3_-exposed mice and human bronchial epithelial cells administrated with O_3_ or H_2_O_2_. Indeed, repeated O_3_ exposure increased TRPC6 protein expression in lungs (Fig. 4a). Lung section IHC also showed an enhanced TRPC6 protein expression after O_3_ exposure, mainly localized in bronchial epithelial cells and macrophages (Fig. 4b). As shown in Fig. 4c, d, TRPC6 protein expression was increased and reached a maximum at 6 h by O_3_ (100 ppb) exposure or H_2_O_2_ (100 μM) stimulation in 16HBE cells. In view of reports highlighting the differences between primary cells with hTERT or viral genes transduced human cells^28,29^, primary bronchial epithelial cells (HBEpiCs) were employed in parts of our study to further confirm the results obtained from 16HBE cells. Consistently, in both 16HBE cells and HBEpiCs, the expression of TRPC6 protein located in both cytomembrane and cytoplasm was upregulated after O_3_ (100 ppb) or H_2_O_2_ (100 μM) exposure for 6 h (Fig. 4e, f).

### TRPC6 mediates H_2_O_2_-induced increase of intracellular calcium in human bronchial epithelial cells

**Fig. 5:**
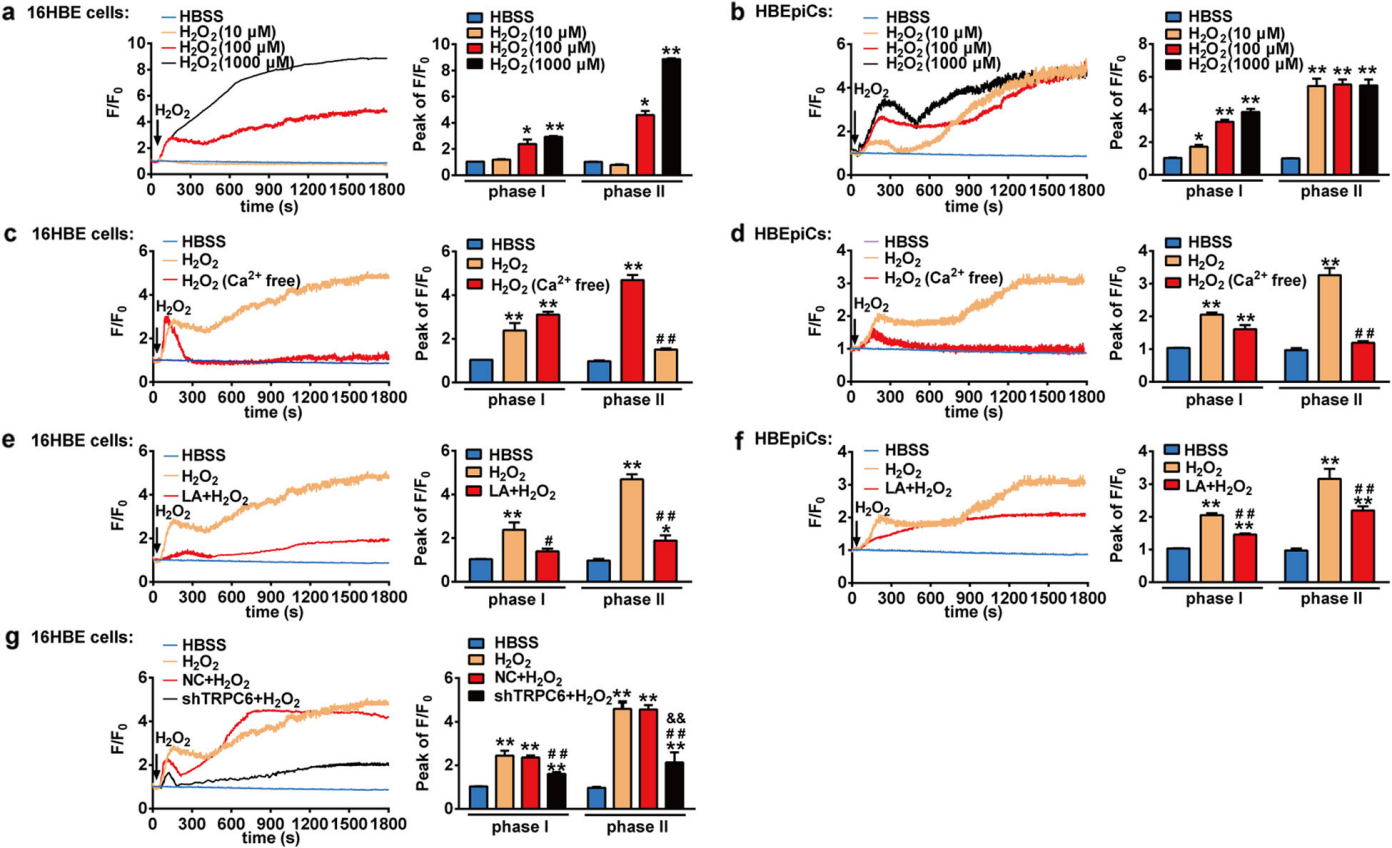
Role of TRPC6 in H_2_O_2_-induced [Ca^2+^]_i_ increase in human bronchial epithelial cells. 16HBE cells or primary HBEpiCs were loaded with the Ca^2+^ indicator Fluo-4/AM (5 μM) in Hanks’ solution for 30 min to measure [Ca^2+^]_i_. The [Ca^2+^]_i_ in the cells was expressed as a pseudo-ratio value of the relative fluorescence intensity (F/F_0_) (Left). Bar graph shows the mean peak value of F/F_0_ traces (Peak of F/F_0_) of the experiments (Right). **a**, **b** 16HBE cells (**a**) and primary HBEpiCs (**b**) were exposed to variable concentrations of H_2_O_2_ (0, 10, 100, 1000 μM). ^*^P < 0.05 or ^**^P < 0.01 compared with HBSS control group. **c**, **d** 16HBE cells (**c**) and primary HBEpiCs (**d**) incubated with or without Ca^2+^-free bath solution (100 μM EGTA) were exposed to H_2_O_2_ (100 μM). ^**^P < 0.01 compared with HBSS control group, ^##^*P* < 0.01 compared with H_2_O_2_ group. **e**, **f** After pretreatment with or without Larixyl Acetate (LA, 10 μM) for 30 min, 16HBE cells (**e**) and primary HBEpiCs (**f**) were exposed to H_2_O_2_ (100 μM). ^*^P < 0.05 or ^**^P < 0.01 compared with HBSS control group, ^#^*P* < 0.05, ^##^*P* < 0.01 compared with H_2_O_2_ group. **g** After NC or shTRPC6 infection, 16HBE cells were exposed to H_2_O_2_ (100 μM). ^**^P < 0.01 compared with HBSS control group, ^##^*P* < 0.01 compared with H_2_O_2_ group, ^&&^*P* < 0.01 compared with NC + H_2_O_2_ group. NC: Negative Control^,^ shTRPC6: shRNA TRPC6. Each trace represents the mean from three independent experiments that were derived from 20 to 40 cells in each single experiment.

Previous studies have shown that oxidative stress leads to the disruption of intracellular calcium homeostasis which is capable of activating signal pathway associated with inflammatory response^7,30^. Therefore, we next aimed to study the effect of H_2_O_2_ on the intracellular calcium homeostasis in bronchial epithelial cells and the role of TRPC6 in this progress. Both 16HBE cells and HBEpiCs were stimulated with different concentrations of H_2_O_2_ and calcium-dependent fluorescence was monitored. Calcium imaging results showed that H_2_O_2_-induced intracellular calcium ([Ca^2+^]_i_) increase in a concentration-dependent manner (Fig. 5a, b)_._ Interestingly, treatment with 100 μM H_2_O_2_ caused a fast [Ca^2+^]_i_ increase (phase Ι) attaining 2.4 ± 0.3 folds within 2 min in 16HBE cells. A slight decline to 2.1 ± 0.1 folds followed and lasted for about 2 min. Beyond this phase, [Ca^2+^]_i_ increased slowly and reached a plateau of 4.6 folds 25 min after stimulation (phase II). 1000 μM H_2_O_2_ caused a faster and more significant [Ca^2+^]_i_ increase in cells which reached the plateau of 8.8 folds 25 min after stimulation (Fig. 5a). Intriguingly, compared with 16HBE cells, HBEpiCs were more sensitive to oxidative stress that 10 μM H_2_O_2_ was capable to trigger this increase (Fig. 5b). To analyze the source of increased [Ca^2+^]_i_, Ca^2+^-free buffer (containing 5 mM EGTA) was applied. Notably, the initial increase of [Ca^2+^]_i_ (phase Ι) induced by H_2_O_2_ (100 μM) was not influenced while the late [Ca^2+^]_i_ increase (phase II) was significantly inhibited (Fig. 5c, d), suggesting that 100 μM H_2_O_2_-induced increase of [Ca^2+^]_i_ was composed by intracellular calcium store release (phase Ι) and extracellular Ca^2+^ influx (phase II), mainly the latter. Moreover, both the phase Ι and phase II of the H_2_O_2_-induced [Ca^2+^]_i_ increase were attenuated by pretreatment with the TRPC6 inhibitor LA (10 μM) and the inhibition of phase II was more salient (Fig. 5e, f). Similarly, knockdown of TRPC6 markedly inhibited 100 μM H_2_O_2_-induced [Ca^2+^]_i_ increase in 16HBE cells (Fig. 5g). Owing to the relatively short lifespan of primary HBEpiCs, which limits total number of times of subculturing, the experiment in the effect of shTRPC6 on H_2_O_2_-induced [Ca^2+^]_i_ increase was unable to carry on. Taken together, these results show that TRPC6 mediates H_2_O_2_-evoked increase in [Ca^2+^]_i_ via the release of intracellular calcium store and the influx of extracellular Ca^2+^.

### O_3_ amplifies H_2_O_2_-triggered increase of [Ca^2+^]_i_ via TRPC6 in human bronchial epithelial cells

**Fig. 6:**
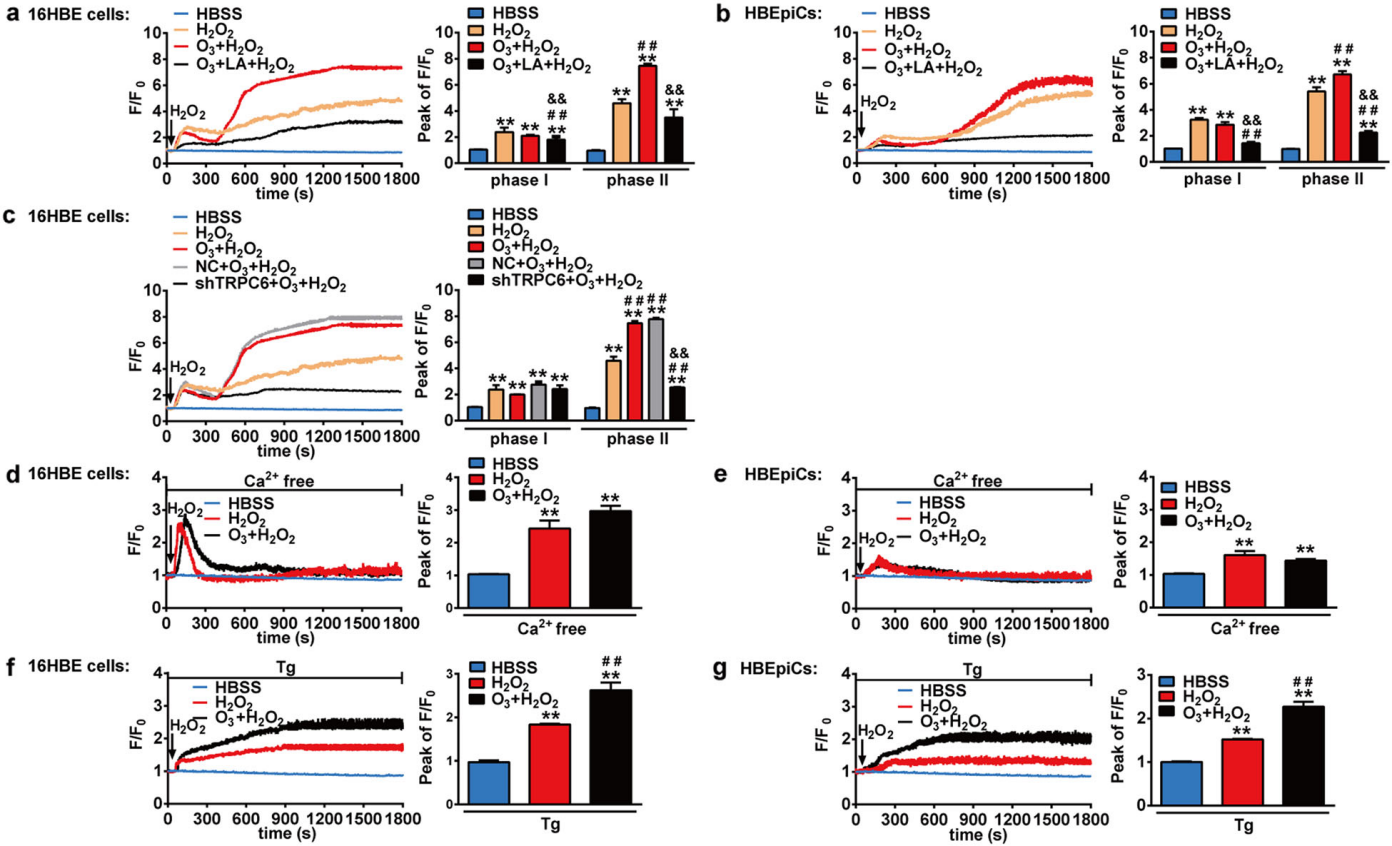
Role of TRPC6 in O_3_ augmenting H_2_O_2_-induced [Ca^2+^]_i_ increase in human bronchial epithelial cells. 16HBE cells or primary HBEpiCs were loaded with the Ca^2+^ indicator Fluo-4/AM (5 μM) in Hanks’ solution for 30 min to measure [Ca^2+^]_i_. The [Ca^2+^]_i_ in the cells was expressed as a pseudo-ratio value of the relative fluorescence intensity (F/F_0_) (Left). Bar graphs show the mean peak value of F/F_0_ traces (Peak of F/F_0_) of the experiments (Right). **a**, **b** After exposure to air or O_3_ (100 ppb) for 6 h, 16HBE cells (**a**) and primary HBEpiCs (**b**) were pretreated with LA (10 μM) for 30 min and then stimulated with H_2_O_2_ (100 μM). ^**^P < 0.01 compared with HBSS control group, ^##^*P* < 0.01 compared with H_2_O_2_ group, ^&&^*P* < 0.01 compared with O_3_ + H_2_O_2_ group. **c** After NC or shTRPC6 infection, 16HBE cells were exposed to air or O_3_ (100 ppb) for 6 h and then stimulated with H_2_O_2_ (100 μM). ^**^P < 0.01 compared with HBSS control group, ^##^*P* < 0.01 compared with O_3_ + H_2_O_2_ group, ^&&^*P* < 0.01 compared with NC + O_3_ + H_2_O_2_ group. **d**, **e** After exposure to air or O_3_ (100 ppb) for 6 h, 16HBE cells (**d**) and primary HBEpiCs (**e**) were incubated with or without Ca^2+^-free bath solution containing 100 μM EGTA and then stimulated with H_2_O_2_ (100 μM). ^**^P < 0.01 compared with HBSS control group, ^##^*P* < 0.01 compared with H_2_O_2_ group. **f**, **g** After exposure to air or O_3_ (100 ppb) for 6 h, 16HBE cells (**f**) and primary HBEpiCs (**g**) were incubated with or without thapsigargin (Tg, 2 μM) and then stimulated with H_2_O_2_ (100 μM). ^**^P < 0.01 compared with HBSS control group, ^##^*P* < 0.01 compared with H_2_O_2_ group. NC: Negative Control, shTRPC6: shRNA TRPC6. Each trace represents the mean from three independent experiments that were derived from 20 to 40 cells in each single experiment.

We then speculated that O_3_ exposure made the cells more sensitive to oxidative stress which resulted in more serious inflammatory responses and oxidative injury. As shown in Fig. 6a, b, O_3_ (100 ppb) exposure for 6 h further amplified H_2_O_2_-triggered [Ca^2+^]_i_ increase in 16HBE cells and primary HBEpiCs, displayed as augment of the phase II but no change in the phase Ι, and both phase Ι and phase ΙΙ were significantly abolished by pretreatment with LA (10 μM) for 1 h. The phase II of H_2_O_2_-induced [Ca^2+^]_i_ increase after O_3_ (100 ppb) exposure was inhibited by shTRPC6 in 16HBE cells (Fig. 6c). These results suggest that TRPC6 has a major role in O_3_-induced augmenting sensitivity to oxidative stress in bronchial epithelial cells.

Then we sought to further address the source of 6-h O_3_ exposure-induced augment of [Ca^2+^]_i_ increase. By incubating cells with Ca^2+^-free bath solution containing 100 μM EGTA, we found that O_3_ exposure made no difference to H_2_O_2_-induced release of intracellular calcium storage (Fig. 6d, e) but enhanced the influx of extracellular Ca^2+^ when the intracellular calcium storage was depleted by pretreatment with thapsigargin (Tg, 2 μM) (Fig. 6f, g). Therefore, O_3_-amplified sensibility to oxidative stress via TRPC6 is mainly dependent on enhancing the influx of extracellular Ca^2+^ in the bronchial epithelial cells.

### MAPK signal pathway contributes to oxidative stress-induced inflammatory response in bronchial epithelial cells

**Fig. 7:**
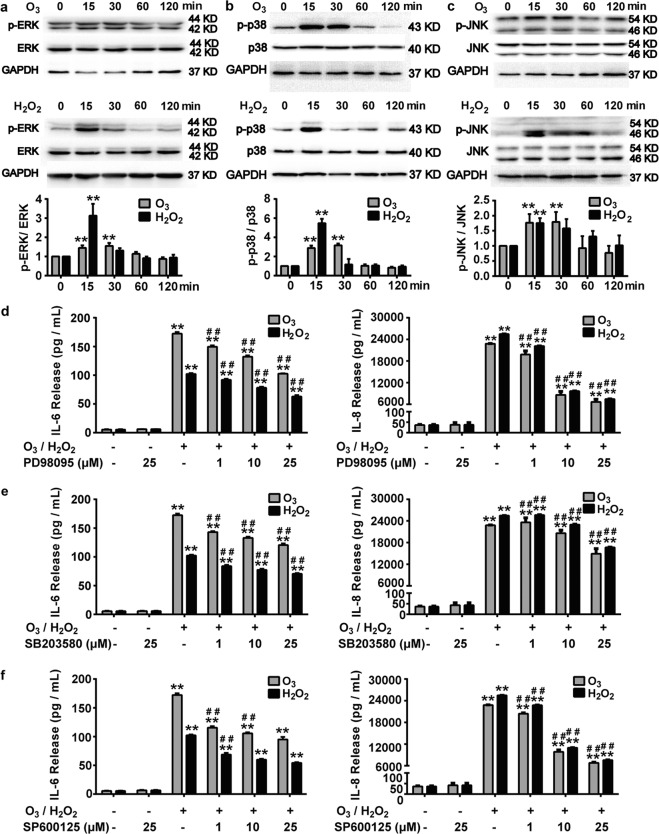
Role of MAPK signal pathway in oxidative stress-induced inflammatory response in bronchial epithelial cells. **a**–**c** Western blot analysis of phosphorylation protein expression of ERK (**a**), p38 (**b**) and JNK (**c**) after 16HBE cells were stimulated with O_3_ (100 ppb) or H_2_O_2_ (100 μM) for 0, 15, 30, 60, 120 min. ^**^P < 0.01 compared with 0-min group. **d**–**f** After pretreatment with or without PD98059 (1, 10, 25 μM) (**d**), SB203580 (1, 10, 25 μM) (**e**) or SP600125 (1, 10, 25 μM) (**f**) for 1 h, 16HBE cells were stimulated with O_3_ (100 ppb) for 6 h followed by continuing culture for another 24 h or stimulated with H_2_O_2_ (100 μM) for 24 h. Release levels of IL-6 and IL-8 were detected. Data represent the mean ± SEM, *n* = 5. ^**^P < 0.01 compared with Control group, ^##^*P* < 0.01 compared with H_2_O_2_ or O_3_ group.

It has been established that MAPK (ERK, p38, JNK) was involved in oxidative stress-induced inflammatory response in lungs^31^. Thus we investigated whether the inflammatory response in this research was mediated by MAPK signal pathway. Results unveiled that after exposure to O_3_ (100 ppb) or H_2_O_2_ (100 μM) for 15 or 30 min, the phosphorylation levels of ERK1/2, p38 and JNK signaling pathways were significantly increased in 16HBE cells (Fig. 7a–c). Pretreatment with PD98059 (ERK inhibitor), SB203580 (p38 inhibitor) or SP600125 (JNK inhibitor) inhibited O_3_ or H_2_O_2_-augmented release of inflammatory factors IL-6 and IL-8 in 16HBE cells in a dose-dependent manner (Fig. 7d–f), suggesting these MAPK signals participated in O_3_ or H_2_O_2_-induced inflammatory response. These data demonstrate that MAPK signal pathway is responsible for oxidative stress-induced inflammatory response in bronchial epithelial cells.

### TRPC6 is required for the oxidative stress-induced activation of ERK pathway

**Fig. 8:**
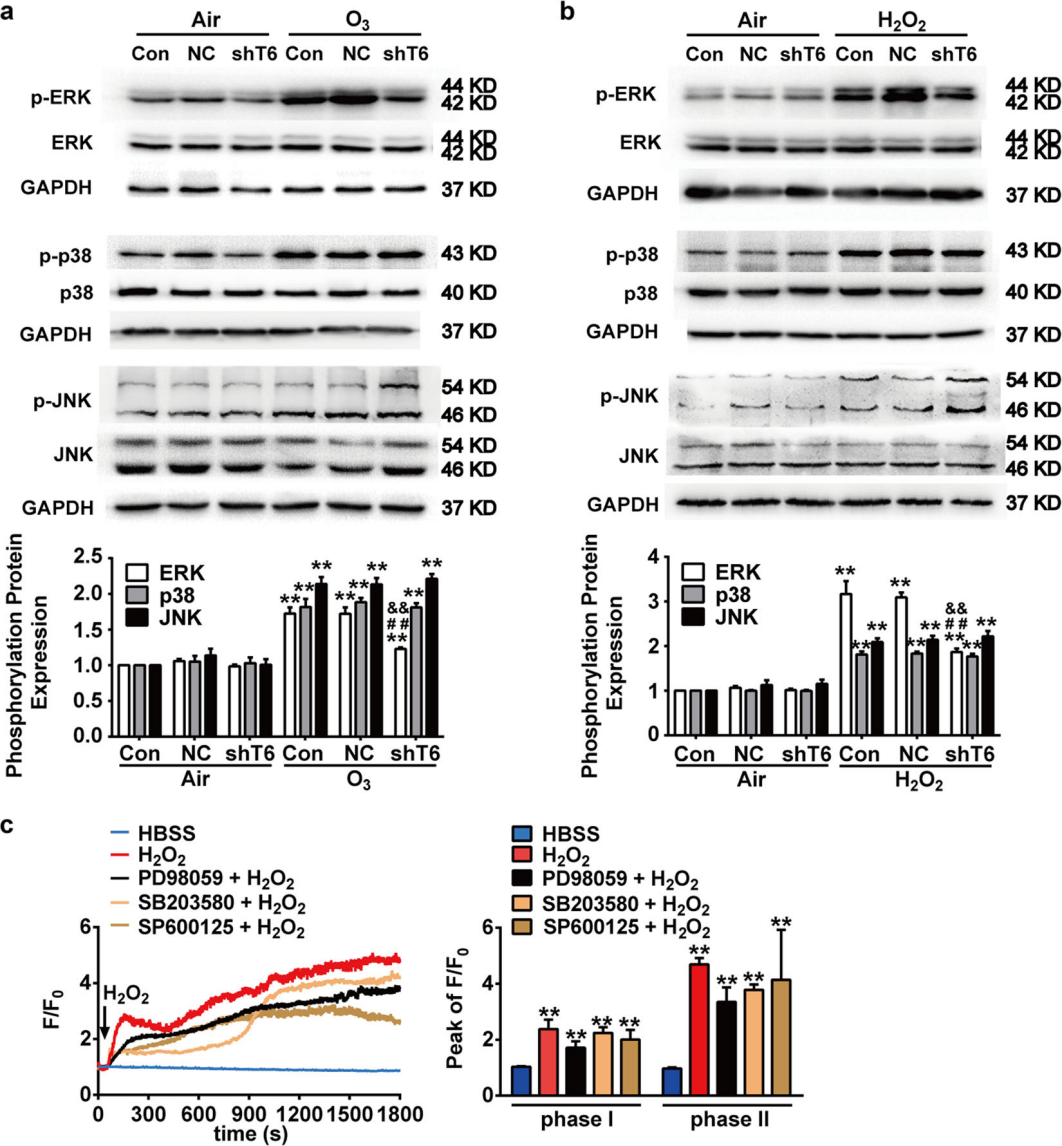
Role of TRPC6 in oxidative stress-induced activation of MAPK signal pathway. **a**, **b** After NC or shTRPC6 infection, 16HBE cells were exposed to O_3_ (100 ppb) for 30 min (**a**) or H_2_O_2_ (100 μM) for 15 min (**b**) respectively. Phosphorylation levels of ERK, p38, JNK were analyzed by western blot. Data represent the mean ± SEM, *n* = 5. ^**^P < 0.01 compared with Control group, ^##^*P* < 0.01 compared with O_3_ or H_2_O_2_ group, ^&&^*P* < 0.01 compared with NC + O_3_ or NC + H_2_O_2_ group. **c** 16HBE cells were loaded with the Ca^2+^ indicator Fluo-4/AM (5 μM) in Hanks’ solution for 30 min to measure [Ca^2+^]_i_. The [Ca^2+^]_i_ in the cells was expressed as a pseudo-ratio value of the relative fluorescence intensity (F/F_0_) (Left). Bar graph shows the mean peak value of F/F_0_ traces (Peak of F/F_0_) of the experiments (Right). After pretreatment with or without PD98059 (25 μM), SB203580 (25 μM) or SP600125 (25 μM) for 1 h, 16HBE cells were stimulated with H_2_O_2_ (100 μM). **P < 0.01 compared with HBSS control group. Each trace represents the mean from three independent experiments that were derived from 20 to 40 cells in each single experiment. NC: Negative Control, shT6: shRNA TRPC6.

We next investigated the role of TRPC6 in oxidative stress-induced activation of MAPK pathways. Deficiency of TRPC6 by shTRPC6 significantly reversed O_3_ or H_2_O_2_-induced increase of phosphorylation levels of ERK but made no difference to those of p38 and JNK (Fig. 8a, b), indicating that TRPC6-mediated oxidative stress-induced activation of ERK pathway rather than p38 or JNK in bronchial epithelial cells. Additionally, pretreatment with MAPK inhibitors (PD98059, SB203580, or SP600125) did not affect the H_2_O_2_-induced [Ca^2+^]_i_ increase (Fig. 8c), which suggested that TRPC6-mediated [Ca^2+^]_i_ increase was not dependent on the MAPK pathways. Taken together, these experiments suggest that ERK acts as the downstream of TRPC6 in oxidative stress-induced inflammatory response.

## Discussion

The major findings of our study demonstrated for the first time that TRPC6 acted as an oxidative stress sensor in bronchial epithelium and mediated oxidants-induced inflammatory responses via activating ERK. Exposure to oxidizing air pollutants (e.g. O_3_) and the following increase of ROS in lungs (e.g. H_2_O_2_) are key causative factors in the development of chronic inflammatory respiratory diseases. Here we found that O_3_ exposure to mice motivated severe airway inflammation and potentiated the expression of TRPC6 protein in lungs, especially in bronchial epithelium and alveolar macrophages. Utilizing TRPC6^−/−^ mice and TRPC6-selective inhibitor, we found that TRPC6 contributed to O_3_ inhalation-induced airway inflammation. In vitro experiments we confirmed the requirement of TRPC6 for oxidative stress-induced inflammatory responses and the upregulation of the TRPC6 protein expression by O_3_ or H_2_O_2_ stimulation in human bronchial epithelial cells. Furthermore, H_2_O_2_-triggered [Ca^2+^]_i_ increase composing of the release of intracellular Ca^2+^ store and influx of extracellular Ca^2+^ was mediated by TRPC6 channels. Importantly, O_3_ exposure enhanced the influx of extracellular Ca^2+^ triggered by H_2_O_2_, which was abolished by TRPC6 knockdown or blockage. Moreover, MAPK signal pathway (ERK, p38, JNK) was responsible for the inflammatory response and ERK pathway acted as the downstream of TRPC6 in these experiments. Therefore, we concluded that TRPC6 regulated oxidative inflammatory responses induced by O_3_ or H_2_O_2_ through activating ERK pathway.

It is well known that exposure to O_3_ induces oxidative injury to the respiratory tract, causes inflammatory responses and triggers clinical symptoms of a series of chronic respiratory disease such as asthma, bronchitis and COPD^32,33^. However, the underlying mechanisms of O_3_-induced oxidative injury have not yet been fully defined. TRPC6, a lipid-dependent membrane protein acting as non-selective cation channels conducting Na^+^ and Ca^2+^, is highly expressed in the lung and most studied in pulmonary diseases among TRPC channels^34,35^. We previously reported that TRPC6 channels contribute to LPS-induced inflammatory response in human bronchial epithelial cells, which implies that TRPC6 may be a therapeutic target in bronchial epithelial inflammation^36^. It has been well described in many cell types and tissues that the activity of TRPC6 channel is redox-sensitive, while it seems to have different phenotype according to different cell types^23,37^. In podocytes, HEK 293T cells and vascular myocytes, ROS not only activates TRPC6 already in the plasma membrane but also upregulates the expression of TRPC6 in the surface^38–42^. Some other studies show that ROS decreases the activity and abundance of TRPC6 protein in mesangial cells^20,43^. However, it has been rarely described whether ROS activate TRPC6 channel, the underlying mechanisms of the following Ca^2+^ transportation and the further effects in respiratory system. Here, we found that O_3_ exposure led to the increase of TRPC6 protein expression in mice lungs (Fig. 4a, b). H_2_O_2_, a diffusible and ubiquitous second messenger, contributes to the pathogenesis of several respiratory disease and their exacerbations^44^. Generated in lungs during oxidative pollutants exposure and more stable to readily penetrate the cells, H_2_O_2_ is adopted in many studies to further investigate the mechanism related to ambient oxidants-induced injury. We found that both O_3_ and H_2_O_2_ enhanced TRPC6 expression in human bronchial epithelial cells (Fig. 4c–e). TRPC6^−/−^ mice or mice pretreated with TRPC6 inhibitor SAR7334 exposed to O_3_ revealed attenuated recruitment of neutrophils, macrophages and lymphocytes into airway, release of inflammatory factor IL-6, IL-8 and TNF-α in BAL fluid and damage of the lungs (Figs. 1, 2). We also confirmed that activation of TRPC6 was required for the release of cytokines after stimulating with O_3_ or H_2_O_2_ in HBECs (Fig. 3). Although we did not detect the levels of ROS in vivo after O_3_ exposure, it is conceivable that oxidative stress generated by O_3_ activates TRPC6 channels and upregulates the expression of TRPC6, which leads to the disruptions of intracellular Ca^2+^ homeostasis and triggers inflammatory response based on the results in vitro (Figs. 3, 5, 6). Surprisingly, the levels of released TNF-α increased after O_3_ exposure in BAL fluid (Figs. 1c, 2c) but it remained unchanged after stimulating with O_3_ or H_2_O_2_ in epithelial cells (Fig. 3a, e). We speculated that TNF-α was secreted by other cells but not bronchial epithelial cells.

Although the functional significance of TRPC6-mediated Ca^2+^ transport system in health and disease has gained widespread attention, its activation mechanism is not completely elucidated yet. H_2_O_2_ has been reported to behave pathophysiologically relevantly with a concentration above 100 μM while a much lower concentration (10 μM) displays a nearly maximal activating effect on TRPC6 channel in HEK293T cells^42^. In the present study, we found that H_2_O_2_ was able to cause concentration-dependently increase of [Ca^2+^]_i_ in 16HBE cells and primary HBEpiCs (Fig. 5a, b), which was mainly resulted from the activation of TRPC6 (Fig. 5e–g). Interestingly, H_2_O_2_-induced increase of [Ca^2+^]_i_ occurred under the concentration of 10 μM in primary HBEpiCs while that occurred under 100 μM in 16HBE cells (Fig. 5a, b), suggesting primary HBEpiCs were more sensitive to oxidative stress than16HBE cells were.

Intracellular Ca^2+^ signal is the most universal and versatile mechanism regulating a wide range of physiological and pathophysiological processes. Store-operated Ca^2+^ entry and receptor-operated Ca^2+^ entry are pharmacologically and molecularly distinctive Ca^2+^ pathways in non-excitable cells. Hence we aimed to illuminate the source of H_2_O_2_-induced increase of [Ca^2+^]_i_ in human bronchial epithelial cells. In this study we presented that H_2_O_2_ triggered [Ca^2+^]_i_ increase in two ways: release from intracellular Ca^2+^ stores and Ca^2+^ influx into the cells (Fig. 5c, d). To date, although ample evidence suggests that TRPC6 is a DAG-sensitive receptor-operated Ca^2+^ entry channel^45^, studies about the source of TRPC6-mediated Ca^2+^ entry generate discordant findings^46^. Indeed, we found that TRPC6-mediated [Ca^2+^]_i_ increase after H_2_O_2_ exposure consisted of the release of intracellular Ca^2+^ stores and the influx of extracellular Ca^2+^ since both phase I and phase II of H_2_O_2_-induced [Ca^2+^]_i_ increase were inhibited under the situation of TRPC6 blockage or deficiency (Fig. 5e–g). Moreover, the profound augment of H_2_O_2_-induced influx of extracellular Ca^2+^ by O_3_ exposure was mainly dependent on TRPC6 channels as the potentiated phase II was abolished by TRPC6 blockage or deficiency (Fig. 6a–c). According to our finding that TRPC6 channels mediated the release of intracellular Ca^2+^ stores, we speculated that TRPC6 channels participated in store-operated Ca^2+^ entry although the detailed process still needs further exploration.

MAPK signal pathway is implicated in inflammatory response induced by oxidative stress^31^. Furthermore, previous studies show relationships between the activation of TRPC6 and MAPK signals, which seemed to be context-dependent^47,48^. Here, we found that treatments with H_2_O_2_ or O_3_ evoked the release of inflammatory factors IL-6 and IL-8 via activating MAPKs (ERK, p38, JNK) (Fig. 7). In addition, deficiency of TRPC6 by shTRPC6 significantly reversed the oxidative stress-induced phosphorylation of ERK but made no effect to that of p38 or JNK (Fig. 8a, b). However, pretreatments with MAPKs inhibitors (PD98059, SB203580, SP600125) did not influence the H_2_O_2_-induced increase of [Ca^2+^]_i_ in 16HBE cells (Fig. 8c), excluding the possibility that MAPK signal pathway acted as the upstream of TRPC6. Therefore, it is suggested that in human bronchial epithelial cells oxidative stress-aroused inflammatory response was mediated by TRPC6 through regulating [Ca^2+^]_i_ increase and subsequently activating ERK signal pathway.

In summary, this is the first report demonstrating that TRPC6 is an oxidative stress-sensitive channel which can further mediate inflammatory response via ERK pathway in bronchial epithelial cells. Our result provides a mechanistic understanding of how oxidizing air pollutants lead to airway inflammation. Moreover, we revealed a critical role of intracellular calcium homeostasis in the pathogenesis of such diseases and proposed that targeting TRPC6 might provide a novel therapeutic approach to prevent and treat oxidative stress-induced airway inflammation.

## Materials and methods

### Cell culture

16HBE cells (Jennio Biotechnology, CHN), a transformed human bronchial epithelial cell line were cultured with 10% fetal bovine serum (FBS) (10099141; Gibco, USA) as previously outlined^36^. Normal primary human bronchial epithelial cells (HBEpiCs) (3210; ScienCell, USA) were maintained in Bronchial Epithelial Cell Medium (BEpiCM) (3211; ScienCell, USA) with 1% bronchial epithelial cell growth supplement. Cells were cultured in humidified air with 5% CO_2_ at 37 °C. Prior to O_3_ or H_2_O_2_ (H6520; Sigma-Aldrich, CHN) exposure, cells were equilibrated by medium containing 1% FBS or 0.1% supplement medium.

### Animals

Eight-week-old female wild-type (WT) and TRPC6-deficient (TRPC6^−/−^) mice, on 129SvEv:C57BL/6 J (50:50) crossbred background, were generously provided by Dr. Lutz Birnbaumer (National Institute of Environmental Health Sciences, Research Triangle Park, North Carolina) and Dr. Yizheng Wang (Shanghai Institutes of Biological Sciences, State Key Laboratory of Neuroscience, CHN). The TRPC6 knockout genotype was confirmed by RT-PCR as previously described^24,49^. Mice were maintained under specific-pathogen-free conditions in the Laboratory Animal Center of Guangzhou Medical University. All animal experiments were carried out under the protocol approved by the Institutional Animal Care and Use Committee of Guangzhou Medical University.

### ShRNA experiments to stably silence TRPC6 and quantitative real-time PCR analysis in 16HBE cells

16HBE cells were transfected with lentivectors expressing TRPC6 shRNA (5′-CCGCUAUGAACUCCU-UGAA-3′) or negative control (5′-TTCTCCGAACGTGTCACGT-3′) (Oboi Techonology, CHN). The rate of effective transfection in the cells was monitored by fluorescence microscopy. Over 95% of cells transfected with lentiviral vectors showed red fluorescence in the experimental and NC groups. The stably transfected cells were picked out with puromycin (4 μg/mL) after being cultured with the lentivirus (1.66 × 10E9 TU/mL) at an infection multiplicity of 80 for 24 h. Deficiency of TRPC6 expression was analyzed with real-time RT PCR and western blot.

Total RNAs were obtained from lungs or cultured cells with the TRIzol reagent (15596018; Invitrogen, USA). Real-time PCR was performed with SYBR Premix Taq Kit (DRR081, TaKaRa, CHN) and analyzed with ABI PRISM 7000 Sequence Detection System (Applied Biosystems, USA). All primers were purchased from Invitrogen Corporation (as illustrated in Table 1). The qPCR results were presented as threshold cycle (Ct) value and the relative mRNA quantification was determined using the 2-^∆∆ct^ method GAPDH as the endogenous control and normalized to a control group.

**Table 1:** Sequences of the primers used for quantitative real-time PCR.

Name	Forward primer (5'−3')	Reverse primer (5'−3')	Product size
TRPC6	GGTGAGCCAGTCTGTTGTCA	TATCTGCTCATGGACTCGGA	109 bp
GAPDH	GAAGGTCGGAGTCAACGG	GGAAGATGGTGATGGGATT	221 bp

### O_3_ exposure

Experiments of O_3_ exposure in vivo were performed in reference to similar exposure study^50^. Mice were randomly assigned into different groups (eight mice per group) and placed awake in whole-body Plexiglas exposure chamber (0.55 m wide, 0.75 m long, 0.65 m high) to be exposed to O_3_ (1 ppm) for 3 h. Another identical exposure chamber was applied for air exposure. Exposure was repeated every other day (day 1, 3, and 5) to optimize airway inflammation and did not induce any weight change in the mice (unpublished data). Mice received SAR7334 (HY-15699; MedChem Express, USA), a TRPC6-selective inhibitor, by oral gavage 4 h before O_3_ exposure, as reported by Maier and colleagues that pharmacologically effective concentrations of SAR7334 reached optimum 4 hours after oral administration and maintained for several hours^51^. No animals were excluded from the analysis and no blinding was carried out for animal experiments.

Cells were exposed to O_3_ (100 ppb) with 5% CO_2_ humidified air at 37 °C in the incubator. During exposure, cells were placed on the 3D rocking platform and tilted gently to an angle of 10° from the horizontal to each quarter to ensure direct contact with O_3_. At the end of the exposure, cells were relocated to another incubator with fresh atmosphere. Assays were performed 0-, 12- or 24-hour after the exposure. Experiments of O_3_ exposure in vitro were done referred to similar exposure study^52^. Before O_3_ exposure, cells were pre-treated with TRPC6-selective inhibitor Larixyl Acetate (LA) (02730595; Sigma-Aldrich, CHN), ERK inhibitor PD98059 (9900; CST, CHN), p38 inhibitor SB203580 (5633; CST, CHN) or JNK inhibitor SP600125 (8177; CST, CHN) for 30 or 60 min.

O_3_ were generated by HAILEA model HLO-800 ozonizers (HAILEA, CHN) and its concentration within the chamber or incubator was monitored over the exposing period by ambient-air O_3_ motors (model 106 L; T2B, USA). The mean concentration of O_3_ within the chambers remained 1 ± 0.05 ppm or 100 ± 10 ppb within the incubator during the exposure.

### Measurement of inflammation by collection and analysis of BAL fluid or supernatants of cells

Mice were anesthetized by intraperitoneal injection of pentobarbital sodium (50 mg/kg) 24 h after the last exposure. To collect bronchoalveolar lavage (BAL) fluid, the left bronchus was clamped, the trachea cannulated and then the right lung lavaged three times slowly with 0.6 mL ice-cold phosphate-buffered saline (PBS). BAL fluids were centrifuged to isolate cells from samples. The supernatants were collected and stored at −80 °C till analysis. To quantify total cell numbers, cell pellets were resuspended with 200 μl PBS and multiplied hemacytometer cell counts excluding red blood cells. Differential cell counts stained by Wright-Giema stain set (D10; Jiancheng Bioengineering Institute, CHN) were determined under an optical microscope (EVOS FI; Advanced Microscope Group, USA). At least 200 cells per mouse were counted under ×200 magnification.

The secretion levels of IL-6, IL-8 and TNF-α in the mentioned supernatants as well as cell medium were detected with commercially available enzyme-linked immunosorbent assay (ELISA) kits (NeoBioscience Technology, CHN) according to the instruction manual. Total protein content was determined using a Pierce BCA Protein Assay kit (23225; Thermo Fisher Scientific, USA). The results were expressed as pg/mg protein.

### Histological analysis

Following BAL fluid collection, the non-lavaged lungs were cut out and immediately inflated in fresh 4% paraformaldehyde buffer. Paraffin blocks were prepared from dehydrated tissues and histological sections (5 μm) were stained with hematoxylin and eosin (H&E) for evaluation with a light microscopic (Olympus BX51, JPN).

The severity of lung inflammation in peribronchial and perivascular in H&E sections was scored on a scale ranging from 0 to 3 where 0 means no inflammation; 1 means mild inflammation with only a few inflammatory cells around bronchial or vascular wall and in alveolar space; 2 means moderate inflammation with patchy inflammatory cells infiltration or localized inflammation around bronchial or vascular wall and in alveolar space and less than one-third of lung cross-sectional area involved; 3 means severe inflammation with diffuse inflammatory cells infiltration and more than one-third of the lung area involved. The scale was made referred to similar study^53^.

### Western blot

After different treatments, 16HBE cells as well as the lung tissue were rinsed with ice-cold PBS. Following steps were performed as previously described^36^. Immunoblotting was detected using Immobilon Western Chemiluminescent HRP Substrate (Millipore, USA) and imaged on ChemiDoc XRS + (Bio-Rad, USA). Western blot bands were quantified by ImageJ 1.41 software and expressed as fold compared to control. Primary specific antibodies used were TRPC6 (CST, #16716), p44/42 MAPK (ERK1/2) (CST, #4695) and Phospho-p44/42 MAPK (ERK1/2) (CST, #4370), p38 MAPK 2103(CST, #4671), GAPDH (Proteintech, 60004).

### Immunohistochemistry (IHC)

IHC was performed on paraffin-embedded lung tissue sections following standard methods. Lung sections (5 μm) were deparaffinized, rehydrated, treated for endogenous peroxidase inhibition and antigen retrieval and then incubated overnight at 4 °C with primary anti-TRPC6 antibody (dilution 1:100) (ACC-017; Alomone Labs, Israel), followed by 30-min incubation with Horseradish peroxidase-conjugated secondary antibody (dilution 1:500). Binding was visualized with DAB and counterstained with hematoxylin. Staining images were taken by a confocal laser scanning microscopy (BX51; Olympus, JPN).

### Fluorescence measurement of intracellular free calcium ([Ca^2+^]_i_)

16HBE cells and HBEpiCs grown on confocal dishes were washed three times with fresh HBSS and 200 μl HBSS was added per dish. The fluorescence intensity of Fluo-4 (5 μM, Molecular Probes Eugene, USA) in the cells was recorded by laser scanning confocal microscopy (Leica TCS SP8, GER). Detailed steps were performed as previously described^36^. H_2_O_2_ was used to stimulate the cells when the baseline was stable. The [Ca^2+^]_i_ was expressed as a pseudo-ratio value (F/F_0_) of the actual fluorescence intensity (F) divided by the average baseline fluorescence intensity (F_0_). The Ca^2+^-free bath solution contained 100 μM EGTA and no CaCl_2_. Data from 20 to 40 cells were compiled from a single run, and at least three independent experiments were conducted.

### Analysis of the expression of TRPC6 protein in the cells by immunofluorescence

The cells cultured on confocal dishes were fixed with 4% formaldehyde in 0.1 M PBS for 15 min and washed three times with 0.1 M PBS. Further details were performed as previously described ^36^6. The cells were incubated with primary specific antibodies against TRPC6 (ACC-017; Alomone Labs, ISR) at 4 °C overnight and rewarmed for 30 min subsequently. Following 3 washes with PBS, the cells were incubated with fluorescein isothiocyanate (FITC)-conjugated secondary antibody (Life Technology, USA) for another 1 h. The nuclei were stained by the fluorescent DNA-binding dye 136 4′, 6-diamidino-2-phenylindole dihydrochloride (DAPI) (Roche, CHN) for 15 min. Fluorescence pictures were taken under a confocal laser scanning microscopy (SP8; Leica TCS, GER).

### Statistical analysis

Statistical analysis was performed with SPSS 13.0 software. Data expressed as mean ± SEM represented at least five independent experiments. Statistical significance was determined using Student’s test or one-way analysis of variance followed by ANOVA and post hoc Bonferroni or Dunnett T3 test. Differences were considered statistically significant when the probability value <0.05 or <0.01.

## Supplementary information

Supplementary Figure Legends

Supplementary Figure .S1

Supplementary Figure .S2
