# Correction: TRPC6-dependent Ca^2+^ signaling mediates airway inflammation in response to oxidative stress via ERK pathway

**DOI:** 10.1038/s41419-020-2678-7

**Published:** 2020-06-25

**Authors:** Qingzi Chen, Yubo Zhou, Lifen Zhou, Zhaodi Fu, Chuntao Yang, Lei Zhao, Shuni Li, Yan Chen, Yousen Wu, Zhenwei Ling, Yufeng Wang, Jianrong Huang, Jianhua Li

**Affiliations:** 1 Affiliated Cancer Hospital & Institute; Key Laboratory of Protein Modification and Degradation, School of Basic Medical Sciences, Guangzhou Medical University, Guangzhou, China; 2 Institute of Pediatrics, Guangzhou Women and Children’s Medical Center of Guangzhou Medical University, Guangzhou, China; 3 The Fifth Affiliated Hospital of Guangzhou Medical University, Guangzhou, China

**Keywords:** Ion channels, Molecular biology

Correction to: *Cell Death & Disease*

10.1038/s41419-020-2360-0 published online 5 March 2020

Since online publication of this article, the authors noticed that there was an error in one of the images in Fig. 3e. The right panel of Fig. 3e shows the release of IL-6, IL-8, TNF-α in 16HBE cells after H2O2 stimulation for 0 to 24 h, but the wrong image was used during compilation. The correct image is shown below. The authors apologise for this error.

**Figure:**
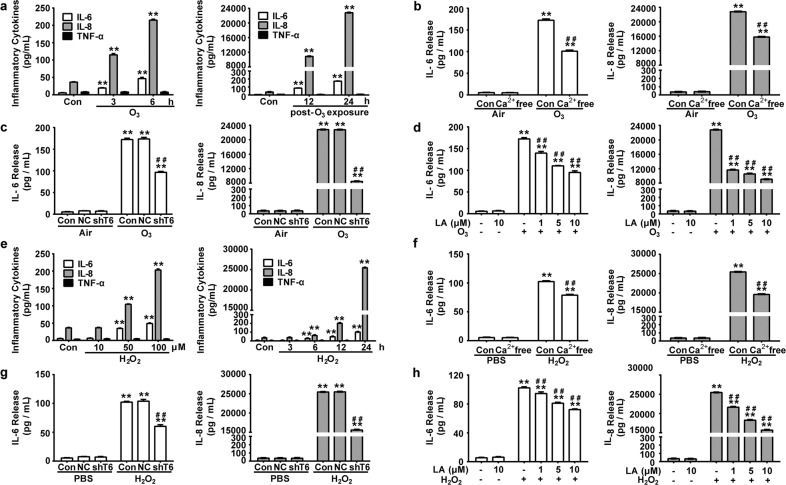
Fig. 3.

