# Supplementary material for: TRPC6-dependent Ca2+ signaling mediates airway inflammation in response to oxidative stress via ERK pathway
Source: Cell Death Dis. 2020 Mar 5;11(3):170. doi: 10.1038/s41419-020-2360-0 (PMC7058000; doi:10.1038/s41419-020-2360-0)
Supplement: Supplementary file 1 — Supplementary Figure Legends [file 41419_2020_2360_MOESM1_ESM.docx]

**Fig. S1. Effect of O_3_ or H_2_O_2_ on the viability of 16HBE cells. a** Viability of 16HBE cells were detected by CCK 8 kit after O_3_ (100 ppb) exposure for 3, 6, 9, 12 h. **b-c** Viability of 16HBE cells were detected by CCK 8 kit after treatment with H_2_O_2_ at 10, 100, 1000 μM concentrations for 12 h **(b)** or after treatment with H_2_O_2_ (100 μM) for 12, 24, 36, 48 h **(c)**. Data represent the mean ± SEM, *n* = 5. ^**^*P* < 0.01 compared with Control group.

**Fig. S2. Deficiency of TRPC6 expression after shTRPC6 infection.** **a-b** TRPC6 expression was detected by real-time RT PCR **(a)** and western blot **(b)**. Data represent the mean ± SEM, *n* = 5. ^**^*P* < 0.01 compared with Control group. NC: Negative Control, shT6: shRNA TRPC6.
