# Supplementary figures and images for: TRPC6-dependent Ca2+ signaling mediates airway inflammation in response to oxidative stress via ERK pathway
Source: Cell Death Dis. 2020 Mar 5;11(3):170. doi: 10.1038/s41419-020-2360-0 (PMC7058000; doi:10.1038/s41419-020-2360-0)

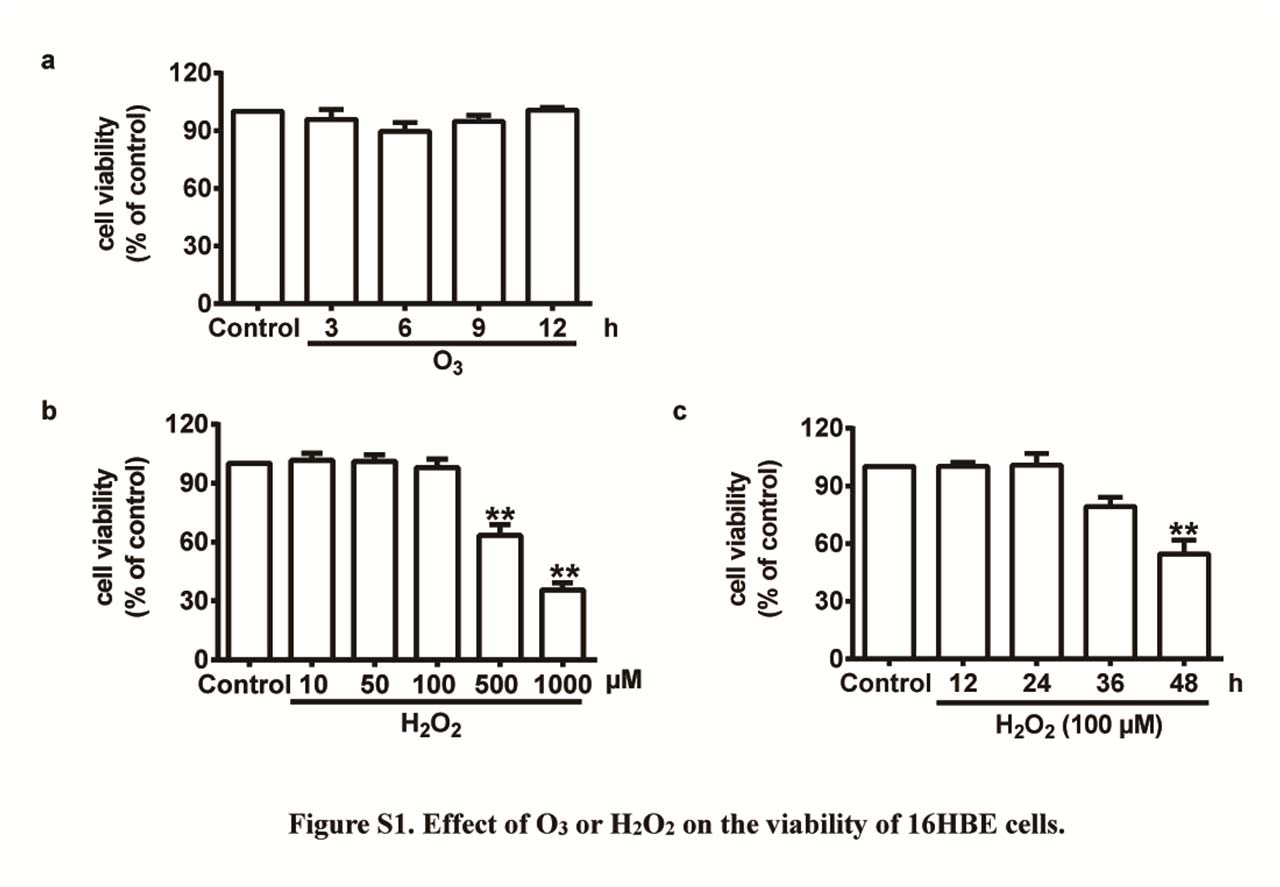

Supplement: Supplementary file 2 — Supplementary Figure .S1 [file 41419_2020_2360_MOESM2_ESM.png]

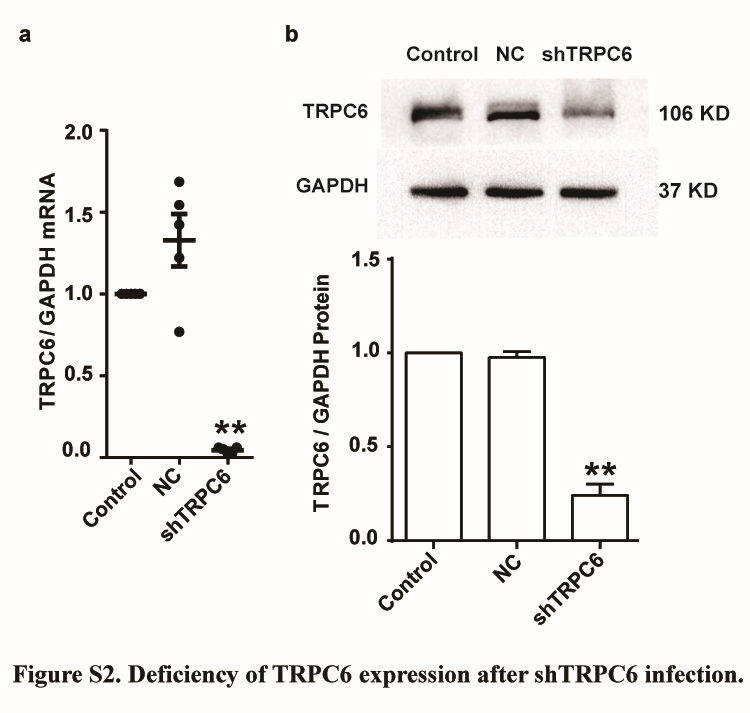

Supplement: Supplementary file 3 — Supplementary Figure .S2 [file 41419_2020_2360_MOESM3_ESM.png]
